# Clinical outcome after entero-enteric anastomosis for Crohn’s disease: a case-control study

**DOI:** 10.1093/ecco-jcc/jjaf163

**Published:** 2025-09-05

**Authors:** Benedetto Neri, Sara Concetta Schiavone, Roberto Mancone, Mariasofia Fiorillo, Antonio Fonsi, Emma Calabrese, Lorenzo Perugini, Gaspare Piccione, Francesco Maria Di Matteo, Irene Marafini, Elisabetta Lolli, Giuseppe Sigismondo Sica, Giovanni Monteleone, Livia Biancone

**Affiliations:** Department of Systems Medicine, Gastroenterology Unit, University “Tor Vergata” of Rome, Rome, Italy; Therapeutic GI Endoscopy Unit, Fondazione Policlinico Universitario Campus Bio-Medico, Rome, Italy; Department of Systems Medicine, Gastroenterology Unit, University “Tor Vergata” of Rome, Rome, Italy; Department of Systems Medicine, Gastroenterology Unit, University “Tor Vergata” of Rome, Rome, Italy; Department of Systems Medicine, Gastroenterology Unit, University “Tor Vergata” of Rome, Rome, Italy; Department of Systems Medicine, Gastroenterology Unit, University “Tor Vergata” of Rome, Rome, Italy; Department of Systems Medicine, Gastroenterology Unit, University “Tor Vergata” of Rome, Rome, Italy; Department of Systems Medicine, Gastroenterology Unit, University “Tor Vergata” of Rome, Rome, Italy; Department of Systems Medicine, Gastroenterology Unit, University “Tor Vergata” of Rome, Rome, Italy; Therapeutic GI Endoscopy Unit, Fondazione Policlinico Universitario Campus Bio-Medico, Rome, Italy; Department of Systems Medicine, Gastroenterology Unit, University “Tor Vergata” of Rome, Rome, Italy; Department of Systems Medicine, Gastroenterology Unit, University “Tor Vergata” of Rome, Rome, Italy; Department of Surgical Science, University “Tor Vergata” of Rome, Rome, Italy; Department of Systems Medicine, Gastroenterology Unit, University “Tor Vergata” of Rome, Rome, Italy; Department of Systems Medicine, Gastroenterology Unit, University “Tor Vergata” of Rome, Rome, Italy

**Keywords:** Crohn’s disease, entero-enteric anastomosis, clinical outcome

## Abstract

**Background and Aims:**

The outcome of Crohn’s Disease (CD) patients with entero-enteric anastomosis (EEA) after small bowel resection is undefined. The primary aim of the present case-control study was to compare the clinical recurrence rate within the first 5 years after surgery in CD patients with small bowel EEA (Cases) versus age-matched patients with ileo-colonic anastomosis (ICA, Controls).

**Methods:**

All CD patients with EEA were matched for age at diagnosis (±5 years) and smoking habits with two Controls with ICA. Inclusion criteria were: (1) age ≥18 years; (2) EEA or ICA for CD; (3) ≥5 years of follow-up after surgery. Exclusion criteria were: (1) missing data; (2) ostomy; (3) stricturoplasty.

**Results:**

The study population included 51 CD patients with EEA and 102 matched Controls with ICA. During the first 5 years after surgery, clinical recurrence and CD-related hospitalizations were more frequent in Cases (34 [66.7%] vs 43 [42.2%], *P* = .007; 25 [49%] vs 23 [22.5%], *P* = .001). During the same period, use of corticosteroids, immunosuppressors, and biologics were also more frequent in Cases (26 [50.9%] vs 18 [17.6%], *P* < .0001; 21 [41.2%] vs 24 [23.5%], *P* = .03; 23 [45.1%] vs 15 [14.7%], *P* = .03). Survival time from clinical recurrence and hospitalization were shorter in Cases (2.36 [1.29-4.35], *P* = .003; 1.71 [1.06-2.77], *P* = 0.02). EEA and use of immunosuppressors before surgery were risk factors for clinical recurrence and CD-related hospitalization at 5 years (2.68 [1.11-6.45], *P* = .02; 2.61 [1.21-5.6], *P* = .01; 2.53 [1.05-6.09], *P* = .03; 2.44 [1.18-5], *P* = .01).

**Conclusions:**

The clinical outcome is more severe in CD patients with EEA than in those with ICA, being associated with a higher rate of clinical recurrence and hospitalization after surgery.

## Graphical Abstract

**Graphical Abstract:**
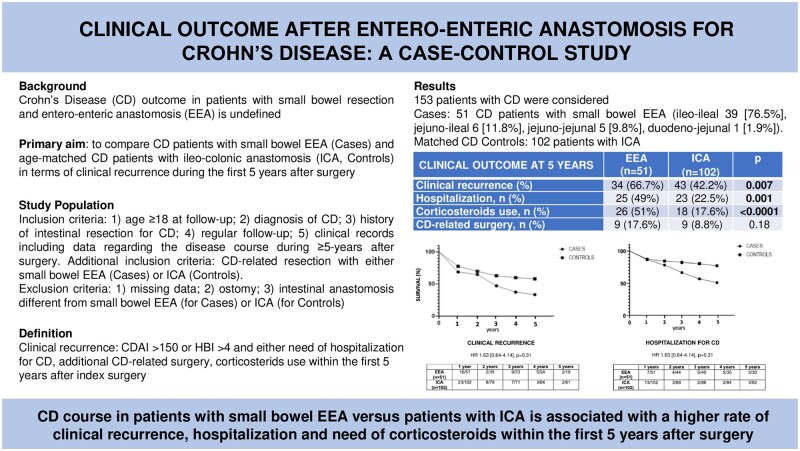

## 1. Introduction

Crohn’s disease (CD) is a chronic-relapsing disease,^1^ requiring surgery in up to 75% of patients.^2–6^ Approximately 35% of patients will require additional surgery within the subsequent 10 years.^2^^,^^3^^,^^7^ Ileo-colonic resection with ileo-colonic anastomosis (ICA) is the most frequent surgical procedure in CD. In these patients, indication for surgery mainly includes obstruction, perforation, abdominal abscess, refractory CD, steroid-dependence, complicated perforating disease, and, less frequently, colonic adenocarcinoma.^8^^,^^9^

Postoperative recurrence (POR) after ileo-colonic resection is evaluated according to both ileocolonoscopy and clinical assessment.^1^^,^^6^^,^^10^ Ileo-colonoscopy is the gold standard for assessing POR after ICA.^11^ The severity of endoscopic recurrence within the first year after ICA has been identified as a significant risk factor for early clinical recurrence.^10^^,^^11^ Cross-sectional imaging, including intestinal ultrasound, and, more recently, histological activity at the peri-anastomotic level are also useful for better defining CD recurrence after ICA.^12^

Smoking is among the most significant risk factors for POR.^13^ Other predictors of early POR after ICA include prior intestinal surgery, small bowel CD, young age at diagnosis, no prophylactic treatments after surgery, and penetrating and perianal CD.^13^

In CD patients at higher risk of early POR, immunomodulatory treatments are recommended.^13^^,^^14^ In these patients, tumor necrosis factor-α inhibitors (TNFi), immunosuppressors (ISS), or other biologics may be used.^13^^,^^14^ Overall, TNFi represent the most effective treatment for preventing POR in selected subgroups of patients.^15^ More recently, a multicenter placebo-controlled trial reported that vedolizumab treatment within 4 weeks from ileo-colonic resection was more likely to prevent endoscopic CD recurrence when compared to placebo.^16^ The same has been suggested for ustekinumab.^17^ However, these lines of evidence refer to therapies for preventing POR after ileo-colonic resection with ICA.

The most common localization of CD is the ileocecal area. However, almost 10%-30% of CD patients show isolated small bowel involvement,^18^^,^^19^ which may require surgical treatment. Surgery mostly includes small bowel resection, although stricturoplasty may be used in subgroups of patients with fibrostricturing CD.^20^

While the clinical course after surgery with ICA in CD is well known, fewer data are available regarding CD course after isolated small bowel resection with entero-enteric anastomosis (EEA). In a previous retrospective study,^21^ we reported a lower frequency of symptomatic recurrence in CD patients with ICA when compared to those with other intestinal resections (37% vs 100%, *P* < .001).

Nevertheless, the clinical outcome of CD patients after small bowel EEA is currently undefined. This issue may be relevant in terms of appropriate assessment and early treatment of POR in these patients, in order to improve the clinical course of CD after small bowel EEA.

On the basis of these observations, the primary aim of the present case-control study was to compare CD patients with EEA after small bowel resection (Cases) versus age-matched CD patients with ICA (Controls) in terms of clinical recurrence rate within the first 5 years after surgery. A secondary aim was to compare Cases and Controls in terms of clinical characteristics and history of CD before surgery. The rates of CD-related hospitalization, CD-related surgery, and use of corticosteroids within 5 years after index surgery were also compared between groups.

## 2. Materials and methods

### 2.1. Study protocol

In a retrospective case-control study, all CD patients with EEA for a disease-related isolated small bowel resection were enrolled (Cases). Each CD patient with EEA was retrospectively matched with two CD patients with ICA (Controls) for a disease-related ileo-colonic resection. Cases and Controls were matched for age at diagnosis of CD (±5 years) and for smoking habits before index surgery.

All patients were in regular follow-up at our tertiary referral IBD center from January 2002 to December 2019.

CD was diagnosed and classified according to standard criteria,^1^ including the Montreal classification.^22^ Data regarding the clinical course of each patient during the first 5 years after index surgery were recorded. For each Case and Control, all data considered were already prospectively reported in clinical records. Major clinical outcomes were considered for both Cases and Controls at 1, 3, and 5 years after index surgery. These included: clinical recurrence (primary endpoint), CD-related surgery, CD-related hospitalization, and need of corticosteroids.

### 2.2. Study population

Inclusion criteria for all CD patients were: (1) age ≥18 years at follow-up; (2) well-defined diagnosis of CD;^1^ (3) history of intestinal resection for CD; (4) regular follow-up at our referral center; and (5) clinical records including data detailing the clinical course of CD during ≥5 years after index surgery. Additional inclusion criteria were CD-related resection with either small bowel EEA (Cases) or ICA (Controls). Exclusion criteria for all patients were: (1) missing data; (2) ostomy; (3) stricturoplasty; and (4) intestinal anastomosis different from EEA (for Cases) or ICA (for Controls).

The demographic and clinical characteristics of all CD patients (Cases and Controls) were recorded in a database including: age, gender, age at CD diagnosis, age at index surgery, time interval from the diagnosis of CD to index surgery (months), smoking habits before and after index surgery (yes vs no/ex), CD duration (years), CD site (ileum, L1; colon, L2; ileum–colon, L3; upper gastrointestinal [GI], L4),^22^ CD behavior (non-stricturing non-penetrating, B1; fibrostricturing, B2; penetrating, B3),^22^ perianal disease (yes/no), prior CD-related surgery (yes/no), extraintestinal manifestations (EIMs) (yes/no, type), treatment with aminosalycilates, corticosteroids, thiopurines, methotrexate, or biologics (yes/no, type, duration), and, when available, ileocolonoscopic or imaging assessment of CD (including intestinal ultrasound, entero-computed tomography [CT] or magnetic resonance imaging [MRI], small bowel follow-through) within 5 years from index surgery. For ileocolonoscopy, recurrence was considered for a Rutgeerts score ≥1.^23^ Medical treatments were separately considered both before index surgery (at any time from the diagnosis of CD to surgery) and after index surgery (within the first 5 years after surgery). The occurrence of clinical recurrence, need of hospitalization, and additional CD-related surgery within the first 5 years after surgery was also recorded. Clinical recurrence was defined according to the Crohn’s Disease Activity Index^24^ (CDAI, activity: >150) and/or Harvey–Bradshaw Index^25^ (activity: HBI > 4), associated with the need of either CD-related hospitalization, surgery, or corticosteroids. Complications after EEA or ICA were defined as early or late (≤30 or >30 days after index surgery) and according to the Clavien–Dindo classification.^26^

### 2.3. Ethical considerations

The study protocol was approved by the local Independent Ethics Committee of the Policlinico “Tor Vergata” of Rome, Italy (Protocol No. 38.23). Patient consent was waived as data were retrospectively collected, the investigation did not add risk for participants, and all data were de-identified.

### 2.4. Statistical analysis

Data were expressed as median [range]. The normal distribution of parametric continuous variables was assessed by using the Kolmogorov–Smirnov test and defined by a *P* > .05. Differences between qualitative and quantitative variables were assessed by the Pearson χ^2^ test, Student’s *t*-test, or Mann–Whitney U-test, as appropriate. Univariate and multivariate logistic regression models were used for assessing risk factors for major clinical outcomes (odds ratio, OR [95% CI]). Factors potentially associated with major clinical outcomes after CD-related surgery were considered for the analysis and only statistically significant variables at univariate analysis were included in the multivariate analysis. Survival from major clinical outcomes was assessed by using Kaplan–Meier curves and the log-rank test (hazard ratio, HR [95% CI]). Statistical significance was considered for all variables in the case of *P* < .05. Statistical analysis was performed using the IBM SPSS statistical software v.26.0.

## 3. Results

### 3.1. Patients with entero-enteric anastomosis for CD

Demographic and clinical characteristics of CD patients with small bowel EEA are summarized in Table 1. Overall, 51 CD patients with EEA were enrolled (13 [25.4%] females). Overall, seven CD patients with EEA did not fulfill the inclusion criteria and they were therefore excluded from the analysis, although showing characteristics comparable to the tested population. In Cases, the median age at diagnosis of CD was 23 [12-80] years, and the median age at surgery was 33 [15-80] years (median time interval from the diagnosis of CD to index surgery 84 [1-360] months). CD localization included the ileum (L1) in 33 (64.7%) patients, and ileum–colon (L3) in 18 (35.3%), while no cases of Crohn’s colitis were observed. Upper GI lesions (L4) were observed in 17 (33.3%) patients. CD phenotype was non-penetrating non-stricturing in one (1.9%) patient, fibrostricturing in 35 (68.6%), and perforating in 15 (29.5%) patients. Perianal disease was observed in five (9.8%) patients before and in 12 (23.5%) patients after small bowel EEA. Active smokers numbered 24 (47%) before index surgery and 22 (43.1%) within the 5 years following small bowel EEA. Before small bowel EEA, 12 (23.5%) patients had a history of ≥1 CD-related surgery.

**Table 1.** Demographic and clinical characteristics of Crohn’s disease (CD) patients with entero-enteric anastomosis (EEA) or ileo-colonic (ICA) anastomosis.

	EEA (*n* = 51)	ICA (*n* = 102)	*P*
Age, years, median [range]	52 [29-85]	54 [23-90]	.42
Age at index surgery, years, median [range]	33 [15-80]	29.5 [15-85]	.39
Age at CD, years, median [range]	23 [12-80]	25 [9-81]	.62
Gender (F), *n* (%)	13 (25.4%)	43 (42.2%)	.06
CD duration at enrollment, median [range]	26 [5-54]	24.5 [5-50]	.5
Time interval from CD diagnosis to index surgery, median [range]	84 [1-360]	21 [1-408]	**.01**
CD characteristics			
A1	8 (15.7%)	13 (12.7%)	.8
A2	35 (68.6%)	74 (72.6%)	.75
A3	8 (15.7%)	15 (14.7%)	.93
L1	33 (64.7%)	70 (68.7%)	.76
L2	0 (0%)	2 (1.9%)	.53
L3	18 (35.3%)	30 (29.4%)	.57
L4	17 (33.3%)	1 (0.9%)	**<.0001**
B1	1 (1.9%)	9 (8.8%)	.2
B2	35 (68.6%)	54 (52.9%)	.09
3	15 (29.5%)	39 (38.3%)	.36
Perianal disease at CD diagnosis, *n* (%)	5 (9.8%)	7 (6.9%)	.74
Perianal disease, from the diagnosis of CD to the end of the study, *n* (%)	12 (23.5%)	17 (16.7%)	.42
Smoking status before surgery (yes), *n* (%)	24 (47%)	46 (45.1%)	.95
Smoking status after surgery (yes), *n* (%)	22 (43.1%)	37 (36.3%)	.51
Extraintestinal manifestations, *n* (%)	9 (17.6%)	24 (23.5%)	.53
Indication for index surgery, *n* (%)			
Fistula/abscess	11 (21.6%)	21 (20.6%)	.94
Obstructive symptoms	35 (68.6%)	69 (67.6%)	.95
Perforation	5 (9.8%)	5 (4.9%)	.41
Bleeding	0 (0%)	1 (0.9%)	.8
Refractory disease	0 (0%)	6 (5.9%)	.49
Intestinal resection before index surgery, (yes), *n* (%)	12 (23.5%)	5 (4.9%)	**.001**
Entero-enteric anastomosis, *n* (%)			
Ileo-ileal	39 (76.5%)	NA	NA
Jejuno-ileal	6 (11.8%)	NA	NA
Duodeno-jejunal	1 (1.9%)	NA	NA
Jejuno-jejunal	5 (9.8%)	NA	NA
Index surgery, year			
Before 2000	10 (19.6%)	37 (36.3%)	.052
After 2000	41 (80.4%)	65 (65.7%)	
Surgical complications			
Early	3 (5.9%)	5 (4.9%)	.89
Late	7 (13.7%)	9 (8.8%)	.74
None	41 (80.4%)	88 (86.3%)	.47
Corticosteroids at time of index surgery (yes), *n* (%)	3 (5.9%)	16 (15.7%)	.14
Corticosteroids within the 1st year before index surgery, (yes), *n* (%)	21 (41.1%)	29 (28.4%)	.16
Conventional immunosuppressors before index surgery, *n* (%)	16 (31.4%)	15 (14.7%)	**.02**
Conventional immunosuppressors at time of index surgery, *n* (%)	9 (17.6%)	5 (4.9%)	**.02**
Biologics at time of index surgery (yes), *n* (%)	3 (5.9%)	5 (4.9%)	.89
Biologics before index surgery (yes), *n* (%)	8 (15.7%)	13 (12.7%)	.8

Abbreviation: NA, not applicable. Bold formatting indicates statistically significant *P* values < .05.

Small bowel EEA was ileo-ileal in 39 (76.5%) patients, jejuno-ileal in six (11.8%), jejuno-jejunal in five (9.8%) and duodeno-­jejunal in one (1.9%) patient. EEA was performed before the year 2000 in 10 (19.6%) patients.

Surgical indications for small bowel EEA included abdominal abscess or symptomatic fistulae in 11 (21.6%) patients, obstruction in 35 (68.6%) patients, and perforation in five (9.8%) patients. Early postoperative complications after surgery occurred in three (5.9%) patients (one anastomotic bleeding, two abdominal abscesses, not requiring additional surgery), while seven (13.7) late complications were observed (five abdominal pain with spontaneous resolution, one anastomotic bleeding, one abdominal abscess). Overall, among the 10 patients with small bowel EEA developing postoperative complications (Table 1), severity according to the Clavien–Dindo classification^26^ was of grade I in six (60%), grade II in two (20%), and grade III in two (20%) patients. Before EEA, 21 (41.1%) patients were treated with systemic corticosteroids, 16 (31.4%) with ISS, and eight (15.7%) with biologics. During the last 3 months before EEA, systemic corticosteroids, ISS, and/or biologics were ongoing in three (5.9%), nine (17.6%), and three (5.9%) patients, respectively. No patients were treated with small molecules during the study period. Additional data of CD patients with small bowel EEA are summarized in Table 1.

### 3.2. Patients with ileo-colonic anastomosis for CD

For each Case with small bowel EEA, two Controls with ICA matched for age at diagnosis of CD (±5 years) and for smoking habits before index surgery were included. Overall, among the 102 Controls (43 [42.2%] females), the median age at diagnosis of CD was 25 [9-81] years and the median age at time of ICA was 29.5 [15-85] years. Active smokers numbered 46 (45.1%) before index surgery and 37 (36.3%) within the 5 years after ICA (Table 1). In Controls, the median time interval between the diagnosis of CD and index surgery was 21 [1-408] months. Surgery was performed before the year 2000 in 37 (36.3%) patients. Surgical indications included abdominal abscess or symptomatic abdominal fistulae in 21 (20.6%) patients, obstruction in 69 (67.6%), perforation in five (4.9%), refractory CD in six (5.9%), and bleeding in one (0.9%) patient. Postoperative complications occurred in 14 (13.7%) patients: early in five (one dehiscence requiring temporary ileostomy, one abscess, one wound infection, one deep vein thrombosis, one severe abdominal pain), and delayed in nine (four abdominal abscesses [one requiring temporary ileostomy], three abdominal pain, two anastomotic bleeding). In the Control group, severity of postoperative complications^26^ occurring in 14 patients was of grade I in nine (64.3%), grade II in three (21.4%), and grade III in two (13.3%) patients.

Before surgery with ICA, systemic corticosteroids were used in 29 (28.4%) patients, ISS in 15 (14.7%), and biologics in 13 (12.7%). Within the 3 months before surgery with ICA, systemic corticosteroids, ISS, or biologics were ongoing in 16 (15.7%), five (4.9%), and five (4.9%) patients, respectively. No patients with ICA were treated with small molecules during the study period. Other characteristics of CD patients with ICA are summarized in Table 1.

### 3.3. Clinical characteristics of CD: comparison between patients with EEA versus patients with ICA

When comparing clinical characteristics between patients with small bowel EEA versus patients with ICA, Cases showed a longer time interval from the diagnosis of CD to index surgery (84 [1-360] vs 21 [1-408] months; *P* = .01). A higher frequency of upper CD lesions was also detected in Cases versus Controls (17 [33.3%] vs 1 [0.9%]; *P* < .0001). The proportion of active smokers was comparable between Cases and Controls (24 [47%] vs 46 [45.1%], *P* = .95 before index surgery and 22 (43.1%) vs 37 [36.3%], *P* = .51 within 5 years after index surgery) (Table 1). A history of any CD-related surgery before index surgery was more frequent in Cases than in Controls (12 [23.5%] vs 5 [4.9%], *P* = .001). ISS use before index surgery was also more frequently observed in Cases versus Controls (at any time: 16 [31.4%] vs 15 [14.7%], *P* = .02; within 3 months from surgery: 9 [17.6%] vs 5 [4.9%], *P* = 0.02).

Rates of early and late postoperative complications were comparable between Cases and Controls (early: 3 [5.9%] vs 5 [4.9%], *P* = 0.89; late: 7 [13.7%] vs 9 [8.9%], *P* = .74, respectively).

According to the study protocol, findings from all Cases and Controls refer to a 5-year follow-up after EEA or ICA. Nevertheless, when considering the entire median follow-up duration after index surgery to the last visit, a shorter follow-up was observed in Cases versus Controls (12 [5-52] vs 15 [5-50] years, *P* = .015).

Additional demographic and clinical characteristics were comparable between Cases and Controls (Table 1).

### 3.4. Clinical outcome within the first 5 years after index surgery: comparison between CD patients with EEA versus patients with ICA

At 3 and 5 years after index surgery, clinical recurrence was significantly more frequent in Cases than in Controls (28 [54.9%] vs 37 [36.3%], *P* = .04 and 34 [66.7%] vs 43 [42.2%], *P* = .007, respectively) (Table 2). Accordingly, a higher frequency of CD-related hospitalization within the first 5 years after small bowel EEA or ICA was observed in Cases than in Controls (25 [49%] vs 23 [22.5%], *P* = .001). By contrast, a comparable proportion of Cases and Controls required additional CD-related surgery at 1, 3, and 5 years after index surgery (1 [1.9%] vs 8 [7.8%], *P* = .27; 4 [7.8%] vs 9 [8.8%], *P* = .91; 9 [17.6%] vs 11 [10.8%], *P* = .35, respectively). POR of the lesions ≤5 years was detected in 89.2% (*n* = 33) of the 37 (72.5%) tested Cases, according to ileocolonoscopy (POR in 1/1), small bowel contrast ultrasonography (SICUS) (POR in 20/21 [95.2%]), entero-CT/MRI (POR in 10/13 [76.9%]), or small bowel follow-through (POR in 2/2 [100%]). Among the 102 Controls, POR was detected in 84% (*n* = 63) of the 75 (73.5%) patients assessed by ileocolonoscopy (POR in 59/69 [84.1%]) or by SICUS (POR in 5/6 [83.3%]).

**Table 2.** Clinical outcome at 1, 3, and 5 years after index surgery: comparison between Crohn’s disease (CD) patients with entero-enteric anastomosis (EEA) versus patients with ileo-colonic anastomosis (ICA).

	EEA (*n* = 51)	ICA (*n* = 102)	*P*
CD-related surgery after index surgery, *n* (%)			
At 1 year	1 (1.9%)	6 (5.9%)	.49
At 3 years	4 (7.8%)	7 (6.9%)	.91
At 5 years	9 (17.6%)	9 (8.8%)	.18
Clinical recurrence			
At 1 year	17 (33.3%)	23 (22.5%)	.21
At 3 years	28 (54.9%)	37 (36.3%)	**.04**
At 5 years	34 (66.7%)	43 (42.2%)	**.007**
Hospitalization, *n* (%)			
At 1 year	7 (13.7%)	13 (12.7%)	.93
At 3 years	15 (29.5%)	17 (16.7%)	.1
At 5 years	25 (49%)	23 (22.5%)	**.001**
Corticosteroids			
At 1 year	14 (27.5%)	8 (7.8%)	**.002**
At 3 years	19 (37.3%)	15 (14.7%)	**.003**
At 5 years	26 (51%)	18 (17.6%)	**<.0001**
Conventional immunosuppressors			
At 1 year	12 (23.5%)	16 (15.7%)	.33
At 3 years	20 (39.2%)	20 (19.6%)	**.01**
At 5 years	21 (41.2%)	24 (23.5%)	**.03**
Biologics			
At 1 year	10 (19.6%)	9 (8.8%)	.09
At 3 years	15 (29.4%)	13 (12.7%)	**.02**
At 5 years	23 (45.1%)	15 (14.7%)	**.03**

Bold formatting indicates statistically significant *P* values < .05.

Systemic corticosteroids at 1, 3, and 5 years after surgery were more frequently required in Cases than in Controls (14 [27.5%] vs 8 [7.8%], *P* = .002; 21 [41.2%] vs 15 [14.7%], *P* = .0006; 26 [50.9%] vs 18 [17.6%], *P* < .0001, respectively). At both 3 and 5 years after index surgery, ISS use was more frequent in Cases versus Controls (20 [39.2%] vs 20 [19.6%], *P* = .01; 21 [41.2%] vs 24 [23.5%], *P* = .03, respectively). Biologics were also more frequently required in Cases (15 [29.4%] vs 13 [12.7%], *P* = .02 and 23 [45.1%] vs 15 [14.7%], *P* = .03 respectively). No other differences were observed between the two groups as summarized in Table 2.

### 3.5. Clinical recurrence and CD-related hospitalization after index surgery in Cases versus Controls

**Figure 1.**
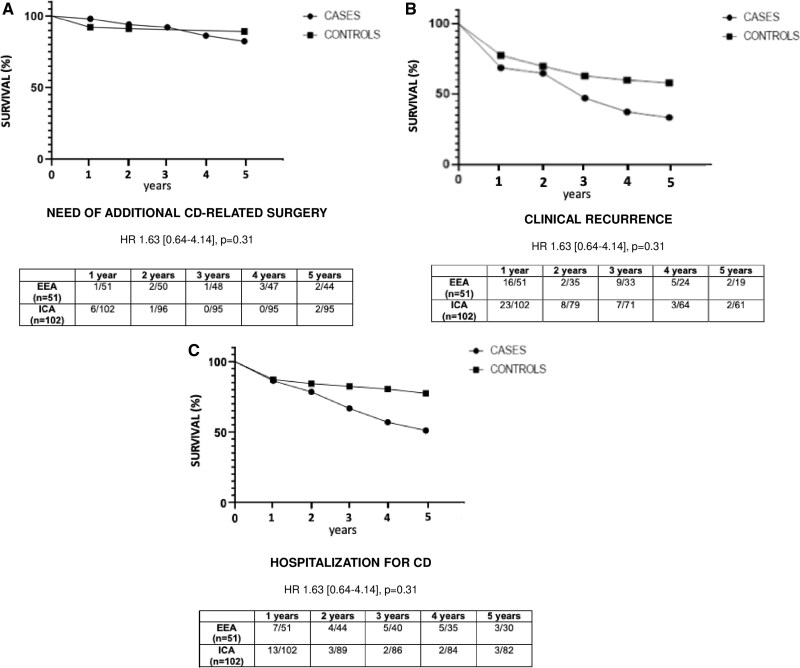
Kaplan–Meier curves showing the survival time from CD-related surgery (A), clinical recurrence (B), and CD-related hospitalization (C) in patients with small bowel entero-enteric anastomosis (EEA) versus patients with ileo-colonic anastomosis (ICA). (A) The survival time from CD-related surgery within the first 5 years after surgery was comparable between patients with EEA and ICA (HR 2.32 [0.75-5.39], *P* = .15). (B) By contrast, a shorter time to survival time from clinical CD recurrence was observed in patients with EEA versus patients with ICA (HR 1.71 [1.06-2.77], *P* = .02). (C) A shorter survival time from CD-related hospitalization was also observed in patients with EEA versus patients with ICA (HR 1.71 [1.06-2.77], *P* = 0.023). Abbreviations: CD, Crohn’s disease; HR, hazard ratio.

Within the first 5 years after index surgery, the survival time from CD-related surgery was comparable in Cases versus Controls (HR 1.63 [0.64-4.14]; *P* = .31) (Figure 1A). By contrast, the survival time from clinical recurrence and CD-related hospitalization was shorter in patients with small bowel EEA than in those with ICA (HR 2.36 [1.29-4.35], *P* = .003 and HR 1.71 [1.06-2.77], *P* = .02, respectively) (Figure 1B and 1C).

The frequency of clinical recurrence, CD-related hospitalization, surgery, and need of corticosteroids within the first 5 years after surgery was comparable between patients undergoing EEA before (*n* = 10) versus after (*n* = 41) the year 2000 (7 [70%] vs 27 [65.8%]; *P* = .9; 4 [40%] vs 21 [51.2%; *P* = .7]; 2 [20%] vs 7 [17.1%; *P* = .8]; 5 [50%] vs 21 [51.2%; *P* = 0.9], respectively). No differences were also observed between patients undergoing ICA (Controls) before (*n* = 38) versus after (*n* = 64) the year 2000 in terms of clinical recurrence, CD-related hospitalization, surgery, and need of corticosteroids ≤5 years after surgery (12 [31.6%] vs 31 [40.4%], *P* = .09; 8 [21.1%] vs 15 [23.4%], *P* = .8; 3 [7.9%] vs 6 [9.4%], *P* = .9; 6 [15.8%] vs 12 [18.7%], *P* = .7, respectively).

### 3.6. Risk factors for clinical recurrence, CD-related hospitalization, and surgery

Among the tested variables, no significant risk factors were identified for CD-related surgery within the first 5 years after index surgery (Table 3). By contrast, at multivariate analysis, prior ISS treatment before surgery and EEA were identified as significant risk factors for CD-related hospitalization within the first 5 years after index surgery (HR 2.68 [1.11-6.45]; *P* = .02 and 2.61 [1.21-5.6]; *P* = .01, respectively) (Table 4). Similarly, ISS use before index surgery and EEA were also significant risk factors for clinical recurrence within the first 5 years after surgery at both uni- and multivariate analysis (HR 2.53 [1.05-6.09]; *P* = .03; 2.44 [1.18-5]; *P* = .01, respectively) (Table 5).

**Table 3.** Risk factors for additional Crohn’s disease (CD)-related surgery within the first 5 years after index surgery in patients with entero-enteric anastomosis (EEA) or ileo-colonic anastomosis (ICA).

	Univariate analysis	
Variable	HR [95% CI]	*P*
Female gender	1.03 [8.15-7.96]	.2
Indication for surgery		
Fistula/abscess	0.63 [0.17-2.31]	.48
Obstructive symptoms	1.11 [0.4-3.1]	.83
Perforation	3.17 [0.74-13.46]	.11
Bleeding	NA	NA
Refractory disease	NA	NA
Surgery before the year 2000	1.2 [0.44-3.25]	.7
CD characteristics		
A1	2.43 [0.78-7.61]	.12
A2	0.93 [0.33-2.61]	.89
A3	0.26 [0.03-2]	.2
B1	1.7 [0.34-8.82]	.5
B2	0.86 [0.33-2.22]	.75
B3	0.98 [0.36-2.63]	.97
L1	1.15 [0.41-3.2]	.78
L2	NA	NA
L3	0.92 [0.33-2.58]	.88
L4	2.12 [0.62-7.25]	.22
Perianal disease	1.36 [0.27-6.74]	.7
Smoking before index surgery	0.34 [0.12-1.01]	.053
Smoking after index surgery	0.64 [0.23-1.78]	.4
Corticosteroids before index surgery	0.86 [0.31-2.41]	.78
Immunosuppressors before index surgery	2.44 [0.88-6.77]	.08
Biologics before index surgery	1.12 [0.3-4.23]	.85
Entero-enteric anastomosis	1.77 [0.68-4.6]	.23
Prior CD-related resection before index surgery	0.87 [0.18-4.14]	.86

Abbreviations: HR, hazard ratio; NA, not applicable.

**Table 4.** Risk factors for Crohn’s disease (CD)-related hospitalization within the first 5 years after index surgery.

	Univariate analysis		Multivariate analysis	
Variable	HR [95% CI]	*P*	HR [95% CI]	*P*
Female gender	1.36 [0.67-2.76]	.38	NA	NA
Indication for surgery				
Penetrating CD	0.67 [0.27-1.63]	.38	NA	NA
Obstructive symptoms	0.89 [0.5-2.19]	.88	NA	NA
Perforation	2.32 [0.64-8.44]	.2	NA	NA
Bleeding	NA	NA	NA	NA
Refractory disease	1.09 [0.19-6.21]	.91	NA	NA
Index surgery before the year 2000	0.63 [0.29-1.37]	.25	NA	NA
CD characteristics				
A1	2.78 [0.69-4.58]	.22	NA	NA
A2	0.72 [0.34-1.52]	.39	NA	NA
A3	0.95 [0.36-2.48]	.91	NA	NA
B1	0.52 [0.11-2.58]	.43	NA	NA
B2	1.01 [0.5-2.01]	.97	NA	NA
B3	1.15 [0.56-2.33]	.7	NA	NA
L1	1.1 [0.52-2.89]	.79	NA	NA
L2	NA	NA	NA	NA
L3	0.99 [0.47-2.07]	.82	NA	NA
L4	2.46 [0.90-6.66]	.07	NA	NA
Perianal disease	1.62 [0.48-5.41]	.42	NA	NA
Smoking before index surgery	1 [0.5-1.99]	.98	NA	NA
Smoking after index surgery	0.93 [0.46-1.89]	.85	NA	NA
Corticosterids before index surgery	1.19 [0.58-2.46]	.62	NA	NA
Immunosuppressors before index surgery	3.56 [1.57-8.06]	**.002**	2.68 [1.11-6.45]	**.02**
Biologics before index surgery	1.11 [0.41-2.95]	.83	NA	NA
Entero-enteric anastomosis	3.03 [1.61-6.78]	**.001**	2.61 [1.21-5.6]	**.01**
Prior CD-related surgery before index surgery	3.68 [1.3-10.38]	**.01**	1.91 [0.59-6.15]	.28

Abbreviations: HR, hazard ratio; NA, not applicable. Bold formatting indicates statistically significant *P* values < .05.

**Table 5.** Risk factors for clinical recurrence of Crohn’s disease (CD) within the first 5 years after surgery with entero-enteric or ileo-colonic anastomosis.

	Univariate analysis		Multivariate analysis	
Variable	HR [95% CI]	*P*	HR [95% CI]	*P*
Female gender	1.54 [0.79-2.98]	.2	NA	NA
Indication for surgery				
Penetrating CD	0.71 [0.32-1.56]	.4	NA	NA
Obstructive symptoms	0.96 [0.48-1.89]	.91	NA	NA
Perforation	2.45 [0.6-9.86]	.21	NA	NA
Bleeding	NA	NA	NA	NA
Refractory disease	0.98 [0.19-5.04]	.98	NA	NA
Surgery before the year 2000	0.52 [0.26-1.06]	.07	NA	NA
CD characteristics				
A1	1.1 [0.43-2.76]	.83	NA	NA
A2	0.53 [0.26-1.09]	.08	NA	NA
A3	2.58 [0.99-6.7]	.051	NA	NA
B1	0.98 [0.27-3.55]	.98	NA	NA
B2	1.41 [0.74-2.69]	.29	NA	NA
B3	0.69 [0.35-1.35]	.28	NA	NA
L1	1.64 [0.83-3.25]	.15	NA	NA
L2	NA	NA	NA	NA
L3	0.68 [0.34-1.35]	.27	NA	NA
L4	2.15 [0.76-6.07]	.14	NA	NA
Perianal disease	0.98 [0.3-3.2]	.98	NA	NA
Smoking before index surgery	1.65 [0.87-3.14]	.12	NA	NA
Smoking after index surgery	1.44 [0.75-2.77]	.27	NA	NA
Corticosteroids before index surgery	1.4 [0.71-2.76]	.32	NA	NA
Immunosuppressors before index surgery	2.97 [1.26-6.99]	**.01**	2.53 [1.05-6.09]	**.03**
Biologics before index surgery	1.37 [0.54-3.48]	.5	NA	NA
Entero-enteric anastomosis	2.74 [1.35-5.54]	**.005**	2.44 [1.18-5]	**.01**
Prior CD-related surgery before index surgery	1.47 [0.52-4.09]	.45	NA	NA

Abbreviations: HR, hazard ratio; NA, not applicable. Bold formatting indicates statistically significant *P* values < .05.

## 4. Discussion

The growing knowledge regarding the pathogenesis of CD gave rise to the development of highly effective treatments for the disease.^27^ However, primary or secondary failures to biologics, CD-related complications, and/or steroid-dependence still require surgery in subgroups of patients.^13^

The natural history of CD after ileo-colonic resection has been extensively investigated.^28^ The role of luminal antigens in the development of POR is strongly supported by “in vivo” evidence showing the efficacy of fecal stream diversion in preventing the POR.^28^ Proven risk factors for POR after ileo-colonic resection include tobacco smoking, prior CD-­related surgery, male gender, perforating disease, and myenteric plexitis.^29^ Whether Kono-S-anastomosis may reduce the rate of POR is under investigation.^29^

Severe endoscopic recurrence after ileo-colonic resection has been shown to predict the clinical outcome of CD.^30^ To prevent complication, ISS or biologics are therefore indicated in this subgroup of patients with early severe endoscopic POR.^14^ Overall, since the introduction of biologics and small molecules for treating CD, a decline of surgery rates has been suggested, although not confirmed.^31^

In contrast to the natural history of CD after ICA, the long-term outcome of CD patients after EEA for isolated small bowel resection is still undefined.^32^^,^^33^ This relates to the less frequent type of anastomosis in CD and to a more difficult and invasive evaluation of the enteric peri-anastomotic area. Accordingly, there is less evidence to indicate the appropriate management and treatment of CD patients with EEA aimed to prevent the POR. The role of endoscopy, which is pivotal in determining the therapeutic strategies in patients with ICA, is only marginal in patients with EEA. Endoscopic assessments of the POR after EEA in CD indeed show technical difficulties also depending on the site of the enteric anastomosis. Small bowel capsule endoscopy (SBCE) is able to visualize the entire small bowel mucosa.^1^^,^^34^ However, in CD patients this technique is burdened by a significant impact risk, thus not representing a diagnostic tool in this setting.^1^ According to current European guidelines,^1^ indication for SBCE in CD is currently represented by patients with symptoms highly compatible with the disease, but no evidence of lesions by using ileocolonoscopy and small bowel imaging.^35^ Indeed, small bowel imaging including intestinal ultrasound may not detect minor postoperative lesions in CD (ie, aphtoid or scattered ulcers), thus limiting the use of these techniques for assessing the early POR after entero-enteric anastomosis. Therefore, whether the detection of early endoscopic recurrence after small bowel EEA may represent a predictor of CD course after surgery is currently undefined. Upper small bowel involvement and other characteristics of CD have been identified as risk factors for a severe disease course after ICA.^19^ However, predictors of a worse outcome after small bowel EEA for CD are currently undefined, preventing optimal treatment strategies after surgery. This is also related to the limited evidence in this regard.

In the present case-control study, we compared clinical characteristics and major outcomes of CD patients with small bowel EEA versus patients with ICA. Cases and Controls were matched for relevant risk factors for a severe CD course after surgery (ie, age at diagnosis of CD and tobacco use). Cases and Controls were not matched for number of surgeries as this parameter was considered among markers of CD severity included in univariate analysis for comparing the two groups. Patients were not compared for number of resections or following first surgeries as the primary aim was to compare the frequency of clinical recurrence within the first 5 years after either EEA or ICA and not after any surgery for CD. The study aimed to assess whether patients with EEA at any time during CD course are at a higher risk of clinical recurrence than patients with ICA.

When compared to Controls, patients with small bowel EEA showed a higher frequency of clinical recurrence and need of CD-related hospitalization, corticosteroids, ISS, and biologics within the first 5 years after surgery. These outcomes appeared not to be related to known risk factors for severe CD course after surgery, including CD phenotype. Smoking was not identified as a significant risk factor for additional CD-related surgery within the first 5 years after index surgery in patients with EEA or ICA. However, smoking before index surgery was at the limit of the statistical significance as a risk factor for additional surgery in both groups (*P* = .053).

The indication for surgery, rate of postoperative complications, and use of biologics before index surgery were comparable between the two groups. Accordingly, in a previous study including young CD patients with extensive and fibrostricturing small bowel disease, a high recurrence risk after small bowel surgery was reported.^33^ However, to our knowledge, no direct comparison between CD patients with EEA and matched patients with ICA in terms of clinical long-term outcome has been reported. The higher rate of both clinical relapse and CD-related hospitalization within the first 5 years after EEA strongly suggests a more severe outcome after surgery in this subgroup of CD patients when compared to patients with ICA. This is further supported by the observation that even before EEA, Cases showed a higher frequency of prior intestinal resection and ISS use than Controls. These data are in agreement with evidence from cohort studies identifying ileal or jejunal CD and the early need of thiopurines as independent risk factors for surgery.^36^^,^^37^ In our study, logistic regression analysis identified EEA and prior ISS use as risk factors for both CD-related hospitalization and clinical recurrence. This further supports that, at least in the tested population, EEA is associated with a more severe postoperative course of CD. The year at time of index surgery showed a wide range in both Cases and Controls, thus leading to possible differences in approach to the management of CD patients after intestinal resection. Therefore, the need of biologics or ISS was not considered as a marker of severe disease postoperatively. Accordingly, the POR of the lesions was searched according to both the type of anastomosis and the different management modalities at time of surgery, thus showing wide interindividual variations. Nevertheless, this information was available for a large majority of the tested population, and the observed rate of POR was in agreement with current evidence in this regard.^13^^,^^28^

The reported findings refer to a maximum follow-up of 5 years after intestinal resection with either EEA (Cases) or ICA (Controls) for CD, as for most of the studies regarding CD recurrence.^23^^,^^30^^,^^38–40^ The observed longer median follow-up duration from index surgery to the last visit in the tested Controls is also in agreement with our reported observation of a longer median time from the diagnosis of CD to index surgery in Cases versus Controls.

Even though small bowel CD is known to be associated with a high frequency of repeat surgery and fibrostricturing behavior,^36^ in the present study these two parameters were comparable between Cases and Controls. A non-statistically significant trend for a higher need of additional surgery within the first 5 years after surgery was observed in patients with EEA vs ICA. Whether the more frequent use of immunomodulators after EEA may have reduced the need for additional surgery in this subgroup of patients may be only hypothesized. Most of the observed differences in terms of clinical outcome in Cases versus Controls were observed after the first 3 years after index surgery. Therefore, it is conceivable that a longer follow-up may detect a greater need for additional surgery.

Among limitations of the study is the availability of data regarding the endoscopic or imaging assessment of the POR of CD lesions only in subgroups of patients with EEA and ICA. Heterogeneity of the site and type of anastomosis in this subgroup of patients may also give rise to differences in terms of CD outcome after EEA. Moreover, in the tested population, the year at time of surgery showed wide interindividual variations, including the pre-biologic era in more than one-fifth of patients. The current therapeutic algorithm for managing the POR was therefore not feasible in a relevant proportion of patients. However, this limitation was observed for both Cases and Controls. In terms of surgical technique, the type of EEA (ie, jejuno-jejunal, jejuno-ileal, ileo-ileal, etc) differed among patients also in terms of extent and site of the resected bowel (proximal vs distal jejunum or ileum). Whether these variables may affect the severity of CD course after EEA could not be assessed in the tested population and, to our knowledge, data at this regard are limited.

The main strengths of the present study include the case-control study design and the tested sample size, when compared with previous studies. Clinical features of the enrolled patients, all in regular follow-up at a referral IBD center, were defined according to the same current standard criteria.^1^^,^^22^ This characteristic of the tested patients should avoid misinterpretations regarding features and risk factors for POR. The observed characteristics of the tested IBD population were comparable to those expected in the general CD population, thus avoiding the risk of bias related to the unintentional selection of subgroups of patients. An additional strength of the study is represented by the study protocol, including Cases and Controls matched (1:2) for major risk factors for severe CD course after surgery.

Overall, the present case-control study provides new “real life” data regarding the long-term outcome of CD patients with EEA. In this subgroup of patients, the present findings support a more severe course after surgery when compared to CD patients with ICA matched for clinical variables. The lack of standard protocols defining the timing and modality for a proper early assessment of POR after EEA may contribute to the observed worse outcome in this subgroup of CD patients after surgery. Knowledge of the natural history of the POR after EAA for CD is required for proper treatment in this subgroup of patients, and this study may shed a light on this. Overall, the present findings suggest that a careful and tight assessment of CD patients is required early after EEA, in order to achieve a timely and appropriate treatment aimed to prevent complications.

## Data Availability

The data underlying this article will be shared on reasonable request to the corresponding author.
